# Assessment of Iodine Concentration in Human Milk from Donors: Implications for Preterm Infants

**DOI:** 10.3390/nu14204304

**Published:** 2022-10-14

**Authors:** Noelia Ureta-Velasco, Kristin Keller, Diana Escuder-Vieco, José C. E. Serrano, Nadia Raquel García-Lara, Carmen R. Pallás-Alonso

**Affiliations:** 1 Department of Neonatology, 12 de Octubre University Hospital, Research Institute i+12, Complutense University of Madrid, 28041 Madrid, Spain; 2 “Aladina-MGU”—Regional Human Milk Bank, 12 de Octubre University Hospital, Research Institute i+12, 28041 Madrid, Spain; 3 Department of Experimental Medicine, Medicine Faculty, Lleida University, 25008 Lleida, Spain

**Keywords:** breast milk, donor human milk, human milk bank, iodine intake, iodine in lactating women, urine iodine concentration, Spain, supplementation

## Abstract

Preterm infants are particularly vulnerable to developing iodine deficiency. Donor human milk (DHM) is the preferred feeding option if the mother’s own milk (MOM) is not available, but information on DHM iodine concentration (DHMIC) is lacking. Hence, we aimed to assess DHMIC to further evaluate the adequacy of iodine provision in preterm infants. Finally, associations that might influence DHMIC were studied. In 113 donors, we measured iodine intake by evaluating dietary records for five consecutive days with the DIAL^®^ Software. From the second day of dietary record, donors provided human milk samples (at least one per day) for four consecutive days. Daily human milk samples were analyzed for DHMIC. A DHMIC ≥ 200 µg/L was considered an adequate iodine content for preterm infants. DHMIC and urine iodine concentration (UIC) were determined using ICP-MS. In our study, 83.2% of donors had a full-term infant. Breastfeeding time range was 1.5–49.4 months. During the dietary record, 55.8% took iodine-containing supplements, providing 40–200 µg/day of iodine. The medians (p25, p75) UIC and DHMIC were 112.4 (75.8, 160.1) and 148.5 (97.6, 206.1) µg/L, respectively. In this iodine-sufficient population, 70% had a DHMIC of <200 µg/L. Donors’ intake of iodine-containing supplements was associated with higher DHMIC.

## 1. Introduction

Thyroid hormones regulate enzymatic and metabolic processes in different ways and ensure normal organ development and growth, playing a crucial role in brain development from pregnancy to the age of 2 years [1,2,3]. Since iodine is an essential component for the synthesis of thyroid hormones, an adequate intake of this trace element prevents developmental problems resulting from iodine deficiency disorders [4].

Preterm infants are at a high risk of iodine deficiency because of early deprivation from the maternal supply and limited capacity to retain dietary iodine. Consequently, the dietary iodine requirement is higher in preterm infants than in term infants [5,6]. Moreover, parenteral nutrition restricts preterm infant iodine exposure, as its marginal iodine content is not sufficient to reach an adequate iodine status [7,8,9].

The recommended enteral iodine supply for preterm infants is 11 to 55 µg/kg/day [10]. Iodine balance studies on healthy preterm infants aged about one month indicated that the daily iodine intake required to maintain a positive balance is at least 30 µg/kg [11]. To achieve such an iodine intake, a breast milk iodine concentration (BMIC) of 200 µg/L might be needed [7,12].

Donor human milk (DHM) is considered the preferred feeding option for preterm infants when the mother’s own milk (MOM) is not available [13]. It is known that BMIC varies widely depending on maternal iodine intake and storage, as well as the stage of lactation, with higher concentrations in colostrum than in mature milk [6,14,15]. However, as Perrin et al. [16] pointed out, findings regarding MOM should not be generalized to DHM because, although it preserves many of the same properties, milk collection methods and the impact of milk banking processes (storage, pasteurization, etc.) influence the composition of DHM. Additionally, most donor milk is term milk, as experiences of human milk banks worldwide show [17,18]. Nevertheless, studies on the nutrient composition of DHM are scarce. Regarding DHM iodine concentration (DHMIC), only two studies reported the iodine concentration in DHM [7,19]. Although pasteurization does not seem to significantly influence iodine concentration [19], there is no available information on whether variations in DHMIC depend on donor mother-infant characteristics or iodine-intake-related variables.

Considering the critical situation and the fragility of preterm infants, the lack of information about DHMIC is concerning. Therefore, the aim of our study was to examine human milk iodine concentrations in donors from a regional human milk bank (HMB). Additionally, the adequacy of iodine provision in preterm infants was assessed and baseline characteristics and iodine intake factors that might influence DHMIC were determined.

## 2. Materials and Methods

### 2.1. Participants and Study Design

The present work is part of a larger research project, a cross-sectional study designed to investigate correlations between dietary intake, nutritional status, and the nutrient composition of human milk. It was conducted between May 2017 and February 2020 at the regional HMB “Aladina-MGU” (12 de Octubre University Hospital, Madrid, Spain), a public HMB, where donation is voluntary and altruistic. In this paper, we present only the results concerning iodine because of its important role in preterm development, nevertheless, other nutrients were assessed in the research project and are under evaluation.

Recruitment was by opportunity. The inclusion criteria for the present study were to be a milk donor accepted by our HMB and to be active (last donation less than 2 months ago). Additionally, donors did not have communication barriers, and provided informed consent to participate in the study. As accepted milk donors, these women had a healthy lifestyle and did not suffer from conditions that compromised an adequate health status. In our HMB there is no restriction in the selection of donors based on age criteria or gestational age of the child. Usually, milk donors are accepted from 3 weeks after delivery to ensure that lactation is well established. There is no upper lactation time limit. Women who wish to donate milk after their baby´s death, those following a vegetarian diet with recent normal vitamin B12 blood levels and who commit to taking recommended vitamin B12 supplements and women with well-controlled pregestational, gestational or postpartum hypothyroidism are accepted as donors.

For recruitment, donors were contacted by telephone, and they received detailed information on the objectives of the study and the procedure to be carried out. Written information was also sent by e-mail.

An early morning meeting at the regional HMB was arranged with the women who agreed to participate. At that appointment they provided a fasting sample of the first urine of that morning. If woman had forgotten to collect the urine sample at home, it was obtained at the regional HMB while still fasting. Women were weighed and measured, and their body mass index (BMI) was calculated (Electronic medical scale with BMI function Model 799 with measuring rod Model 220, CE approved—Class III, brand Seca^®^, Hamburg, Germany). All participants completed a food consumption frequency questionnaire as well as a health and socio-demographic data survey that assessed (1) sociodemographic data; (2) perinatal and postnatal information about diet, supplementation, medicine intake, diseases and anthropometric data (e.g., pre-pregnancy weight); (3) information about childbirth and lactation; and (4) the habitual intake of iodized salt. One of the researchers assisted participants in completing the questionnaires. Milk donors were trained on how to complete the dietary record at home and were provided with the necessary material for dietary record and milk collection. Written information and a telephone number for further questions were also provided to the donors.

For the second part of the study, participants chose a period of 5 successive days throughout the 15 days following the first appointment. During 5 days (days 1 to 5) they had to fill in a dietary record. From days 2 to 5 they also had to collect at least a milk sample per day. During breast milk collection, donors were required to report the total volume and the time of expression. The five-day dietary record and all milk samples were delivered to the regional HMB within 15 days after study completion.

The urine and milk samples were processed and frozen at −80 °C at the HMB until they were sent on dry ice to the laboratory (NUTREN-Nutrigenomics Group of the Department of Experimental Medicine at the University of Lleida, Spain) for analysis.

The study procedure was endorsed by the 12 de Octubre University Hospital Clinical Research Ethics Committee (protocol code 15/269) and all donors provided written informed consent, which could be withdrawn at any time. Furthermore, the study was conducted in accordance with the Declaration of Helsinki.

### 2.2. Dietary Study for the Evaluation of Iodine Intake

The participants completed a weighted dietary record during five subsequent days, including a weekend day, following published recommendations on interviewees training and description of consumed portion sizes [20]. Briefly, they were required to record food and beverages as consumed, annotating vitamins and minerals supplements as well as medicines intake and the type of salt used (iodized or not iodized). Donors were instructed to weigh the food and beverages with a kitchen scale and only if this was not possible, to use household measurements. The total iodine intake for each donor was estimated for each recording day using the DIAL-Software^®^ (Tokyo, Japan), a program designed to assess diets and to calculate energy and nutrients provided by foods [21]. Food items not included in the software were created based on the information provided by the participants (i.e., from food packages) or using databases such as the Spanish Food Composition Database [22] or Food Data Central from the U.S. Department of Agriculture [23].

Daily servings of dairy products consumed, iodine intake from food alone (information on food and beverage intake) and total iodine intake (considering information from all sources: diet, iodized salt and supplement intake) were assessed through dietary record evaluation.

The weekly fish consumption was determined with a food frequency questionnaire provided to the donors. They were asked to report how often they ate fish and to estimate the approximate weight of their usual servings using a photographic atlas [24]. We calculated the total weight of fish consumed weekly and converted it to portions of 150 g, as proposed by the Spanish Society of Community Nutrition [25].

### 2.3. DHMIC

Breast milk samples were collected at home for four consecutive days, starting one day after the first dietary record. Each time participants expressed milk, they realized a full milk expression from one or both breasts, as is required for normal donation. A full milk expression was achieved when milk stopped flowing, although if this did not happen, we suggested that donors should finish milk expression after 20 min. In accordance with donor routines, a schedule was not provided. Donors took 25 mL from each full expression with a sterile syringe and deposited the sample in a sterile feeding bottle, upon which they specified the date and time of sampling and the total volume expressed. The rest of the milk could be used for donation or for their own children. If mothers expressed more than once a day for donation, they were asked to take a sample from each expression, following the same instructions. We advised the participants to store samples at home at least −18 °C and bring them to the hospital within the next 15 days. Donors transported the milk samples in portable coolers with cold packs and delivered them to the regional HMB frozen, where they remained frozen at −20 °C until processing. Then, we thawed the bottles in a 40 °C water bath and homogenized the milk. In case there were more than one milk sample per day, the milk expressed on the same day was pooled, resulting in a single daily sample. From each day’s sample, 20 aliquots of 1 mL each were taken and placed in Eppendorf tubes. All resulting aliquots were labelled with donor’s identification (ID) and the day of expression, and they were stored at −80 °C.

In the laboratory, the milk samples were thawed for analysis by heating them in a thermoblock at 37 °C for 15 min. This allowed both water-soluble and fat-soluble fractions to be obtained with pipetting. The DHMIC was determined by inductively coupled plasma mass spectrometry (ICP-MS) following the methodology described by Huynh et al. [26]. Briefly, 1 mL of homogenized breast milk was diluted in a mild alkali solution (8% tetramethylammonium hydroxide solution). The samples were then digested at 90 °C and filtered prior to ICP-MS (Agilent 7500ce ICPMS system, Agilent Technologies Inc., Santa Clara, CA, USA).

### 2.4. Urine Iodine Concentration (UIC) and Urine Iodine Creatinine Ratio (UICR)

The urine samples were received at the regional HMB at room temperature, transferred into test tubes and frozen at −80 °C.

Prior to analysis, the urine samples were thawed slowly under refrigeration overnight. Iodine content was directly determined using ICP-MS (Agilent 7500ce ICPMS system, Agilent Technologies Inc.) after a 20-fold dilution adding 4.75 mL of aqueous solution containing 2% 1-butanol, 0.05% EDTA, 0.05% Triton X-100 and 1% NH4OH to 0.25 mL of urine. Creatinine content was determined using the Jaffé colorimetric kinetic method with a commercial assay kit (Ref 1001110, Spinreact, Barcelona, Spain). We used urinary creatinine levels as a marker of urine dilution, allowing us to normalize the iodine urinary data as reflected by the UICR.

### 2.5. Cut-Off Values for DHMIC, Iodine Intake and Iodine Status

We assumed DHMIC ≥ 200 µg/L to indicate adequate iodine content for preterm infants [7,12].

There is a wide range of iodine intake recommendations for lactating women from many different health organizations. In our study, we used the estimated average requirement of 167 µg/day suggested by Dold et al. [27] and the WHO recommendation of 250 µg/day [28].

For the assessment of the iodine status of our milk donor’s population, a median UIC ≥ 100 µg/L [29] and a median DHMIC ≥ 100 mcg/L were considered an adequate iodine nutrition [6,30].

### 2.6. Statistical Analyses

For each individual, we calculated their iodine intake variables and DHMIC as the average of all measurements taken during five and four consecutive days, respectively. For iodine intake variables, it should be noted that because of missing information, studied variables had different sample sizes. For each variable, the sample size reflected the real number of donors who provided verified information about the consumption of iodized salt and iodine-containing supplements. Normality was assessed with the Shapiro–Wilk test. The median and 25th–75th percentiles (p25, p75) described continuous variables, as well as frequency and percentage categorical variables. The mean ± standard deviation (SD) for DHMIC, UIC, UICR and iodine intake was also used to compare our data with other studies. Regarding the univariate approach, we used Mann–Whitney U tests and Kruskal–Wallis tests to determine between-group differences, and we used Spearman correlations to assess associations between continuous variables. Furthermore, multivariate linear regression models determined the independent predictors of DHMIC (log 10 transformed). We included variables (within-subject effects) considered to be associated with DHMIC in our univariate analysis based on a *p*-value of less than 0.10. Regression models were also adjusted for donor IDs in order to estimate between-subject effects. Iodine supplement use during dietary recording was further analyzed (univariate approach) to identify baseline characteristics promoting its intake using Mann–Whitney U tests, Kruskal–Wallis tests and Fisher’s exact tests. In our analysis, the sample sizes for individual variables reflect missing data. Data analyses were performed using the SPSS 19 software (IBM SPSS Statistics Inc., Chicago, TX, USA). Significance was generally set at *p* < 0.05.

## 3. Results

During the recruitment, 368 donors were contacted by telephone. Among the 114 recruited human milk donors, there was only one loss. A total of 113 donors completed the survey, 94 with full-term infants and 19 with preterm infants. For donors with preterm infants, one had twins and another one had a stillbirth. All were omnivores except 2 donors with full-term infants who were ovo-lactovegetarians. The duration of breastfeeding at the time of the study was highly variable, with a minimum of 1.5 months and a maximum of 49.4 months. Regarding thyroid disorders, 16 (14.2%) participants had well-controlled gestational hypothyroidism and 1 participant had subclinical hyperthyroidism only during the gestation. Four participants had well-controlled hypothyroidism at the time of the study. The baseline characteristics of the 113 donors are shown in Table 1.

All donors who completed the study submitted the dietary record, which enabled us to present data on iodine intake from food alone for all of them (n = 113). However, some participants, although they previously reported current intake of iodine supplement and/or iodized salt in the health questionnaire, they did not include this information in the dietary record and we considered this data as missing (we could not find out if they forgot to note this information or if they really did not take them). Hence, sample size regarding total iodine intake was n = 84 due to the lack of reliable information on the intake of supplements and/or the type of salt consumed during the dietary record of 29 women.

In Table 2, we can observe a median (p25, p75) DHMIC of 148.5 (97.6, 206.1) µg/L which is below 200 µg/L. The DHMIC ranged from 25.5 to 472.4 µg/L. On the other hand, the median UIC exceeded 100 µg/L. Total iodine intake was more than double the iodine intake considering only information about food and beverages.

Inadequacy of DHMIC and iodine intake are presented in Table 3. In total, 70% of the donors showed a DHMIC of less than 200 µg/L. When we consider iodine intake from food only, more than 90% of the donors demonstrated an intake below 167 µg/day (Dold recommendation) and below 250 µg/day (WHO recommendation). However, considering total iodine intake, inadequacy dropped to nearly 30% for Dold recommendation and around 45% for the WHO recommendation.

Mean and standard deviation (SD) are also shown (although the variables distribution was not normal, we present them to make comparisons with published).

Table 4 shows the investigated iodine-related characteristics of the donors. In total, 108 (95.6%) women reported to have consumed iodine-containing supplements during pregnancy. Considering the information given in the health questionnaire, 85.8% (n = 97) of the women took iodine supplements during lactation with 67.3% (n = 76) still taking them currently. However, only 55.8% (n = 63) of the women confirmed this intake in the dietary record. Iodized salt intake reported in the health questionnaire was 78.9% (n = 86/109) although considering the data reported in the dietary record only 62.8% (n = 71) of the women confirmed the iodized salt intake. The median (p25, p75) daily intake of iodized salt was 0.9 (0.70, 1.24) g.

Between-group differences and univariate associations between DHMIC and baseline characteristics, iodine intake variables, UIC, UICR and iodine-related variables are described in Table 5. We found that DHMIC decreased with increased breastfeeding duration whereas donors who reported to breastfeed more than one child had significantly higher DHMIC. A positive correlation was observed with total iodine intake but not with iodine intake from food only. Furthermore, higher DHMIC was found in the iodine-supplemented donor group although not in donors who used iodized salt during dietary record. Additionally, DHMIC was significantly higher in donors who reported consuming three or more portions of dairy products daily compared to donors who consumed less than three.

Table 6 shows the association found by multivariate linear regression between DHMIC and the covariates: ID, breastfeeding duration, gestational age, tandem breastfeeding, UICR, consumption of dairy products per day and iodine supplement use during dietary record. Gestational age and UICR were included in the regression analysis as both showed *p*-values below 0.1 during the assessment of univariate associations. We found that the positive association between DHMIC and iodine supplement use was the only one that remained significant after adjustment. The association between DHMIC and breastfeeding duration did not remain significant after including iodine supplement use.

Table 7 shows differences between the two groups (Yes/No) of iodine supplement use during dietary record and baseline characteristics, iodine intake variables, UIC, UICR and iodine-related variables. Notably, no differences were found between supplemented and no supplemented donors and iodine intake from food only. On the other hand, we observed group differences for iodine supplement use and breastfeeding duration. Only 14.3% of the donors who breastfed for more than 12 months took supplements compared to 39.7% who breastfed from 6 to 12 month or 46.0% who nursed less than 6 months at the moment of dietary record.

## 4. Discussion

The present study reports on iodine concentration in the DHM of 113 donors from Spain. The adequacy of iodine provision in preterm infants was also evaluated, as well as associations between DHMIC and donor baseline characteristics, iodine intake variables, UIR, UICR and iodine-related variables. The median DHMIC observed in our study was 148.48 µg/L. It is noteworthy that the milk of 70% of the studied donors did not reach the iodine concentration of 200 µg/L recommended to meet the needs of preterm infants, despite being an iodine-sufficient population. This percentage was found to be significantly different between the supplemented group (54%) and the non-supplemented group (92.5%).

Iodine in breastmilk has been widely studied and its concentration varies substantially across studies around the world, ranging from 20 to 1000 µg/L [6], but there is scarce scientific evidence about DHMIC [16]. Our DHMIC is within the range of 100–200 µg/L considered optimal for BMIC [6,12] and similar to one of the two studies in which iodine concentration in DHM has been assessed [7,19].

The requirements for iodine are higher in preterm infants than in term infants [12]. Iodine balance studies carried out in Belgium estimated that an iodine intake of 30 μg/kg/day is required to reach a positive iodine balance in healthy preterm infants of approximately one month of age, as compared to 15 μg/kg/day for term infants [31]. Even though they were assessed around three decades ago, and in a country that was iodine-deficient at the time, these values are generally used when referring to adequate iodine intake in neonates [5,11,12,32]. On the other hand, the ESPGHAN Committee on Nutrition, although recommending a daily iodine enteral intake between 11 µg/kg and 55 µg/kg for healthy preterm infants, pointed out that the available data are insufficient to draw firm conclusions [10]. The main nutritional source of iodine in the preterm infant is the enteral feeding, as parenteral nutrition provides very small amounts of iodine (0.5–3 µg/kg/day) [7,8,9]. Considering the results of our study and to take them into clinical practice, with respect to our median DHMIC of 148.5 µg/L, the estimated iodine intake for 1 kg preterm infant receiving a donated milk volume of 175 mL/kg/day would be 26 µg/kg/day. Using the same example but considering the median DHMIC obtained from supplemented donors (182.7 µg/L), the infant would receive 32 µg/kg/day. Bovine-based multi-nutrient fortifiers for human milk in Spain provide 11 or 16.9 µg of iodine per 100 mL of milk. Hence, a preterm baby of 1 kg with fortification at established doses, would obtain 45.3 or 55.6 µg/kg/day considering the median DHMIC found in total donors studied vs. 51.3 or 61.6 µg/kg/day considering the median DHMIC in supplemented donors. In any case, the high variability of iodine concentration in human milk should be taken into account. In our study, the donor with the highest DHMIC showed a 4-day mean of 472.4 mcg/L, which would provide 101.9 or 112.3 µg/kg/day of iodine when considering the fictive preterm infant above with additional fortification. This amount of iodine is above the 100 µg/kg/day established by the FAO/WHO as the iodine upper limit intake [33] and associated with an increased risk of developing subclinical hypothyroidism in the preterm infants [34]. In this sense, excessive iodine intake from breast milk has been reported in Asian preterm infants due to maternal consumption of large amounts of seaweed. In these cases, BMIC up to 8500 mcg/L was determined [34].

A systematic review of iodine in human milk [15] determined factors associated with BMIC, such as iodine intake, supplementation and iodized salt use, as well as breastfeeding duration and UICR rather than UIC. In our study, we carried out not only a univariate approach but also a multivariate linear regression, including all variables found to be associated with DHMIC in our univariate analysis. Iodine supplementation during the dietary record was the only factor that maintained its association with DHMIC after adjusting for the rest of the variables. We were unable to demonstrate a positive association between UIC or UICR with BMIC. Both UIC and BMIC reflect the mother´s iodine intake in the preceding hours. Observational studies that found a positive association between the two parameters, collected concomitantly both types of samples [35,36]. In our study, we collected a single urine sample firstly to assess nutritional iodine status whereas milk samples were collected in a posterior time (days later). This might explain why in our study we did not find an association between the two variables. Interestingly, breastfeeding duration showed a negative relationship with DHMIC; however, it was not independent of iodine supplementation. We found that breastfeeding duration was lower in supplemented donors compared to donors who did not take iodine supplementation, which might indicate a lower percentage of donors using iodine supplementation as breastfeeding extends. Our study showed that the use of iodized salt and iodine pharmacological supplements additionally to food was necessary to reach the recommended intake of iodine. Total iodine intake, determined by considering all sources of iodine, was more than twice the iodine intake observed when considering food alone. However, we were not able to relate iodized salt consumption to DHMIC. The high percentage of donors who took pharmacological iodine supplements may have masked the effect of iodized salt in our population. According to our results, it seems appropriate to recommend that milk donors take pharmacological iodine supplements to increase the iodine content of DHM in order to meet the high needs of preterm infants. Although the long-term clinical benefit of direct iodine supplementation for preterm infants during the neonatal period has not been demonstrated [37], iodine supplementation for breastfeeding mothers might be more effective in reducing iodine deficiency in the child [38]. Regarding the safety of mother´s iodine supplementation, sustained iodine intake during lactation exceeding 500 to 1100 mcg per day should be avoided [28,39].

One major strength of our study is that it focused on studying the iodine concentration of DHM, a fluid on which, to date, little research has been conducted, particularly with regard to its micronutrient concentration. Furthermore, this is the first study to evaluate not only DHMIC but also to determine associations with different factors and characteristics. We performed our research respecting the HMB operation [40], as we did not employ additional donor exclusion criteria or defined schedules for breastmilk expression. Regarding methodological strengths, dietary intake was assessed with a weighted dietary record for five consecutive days, including a weekend day. Additionally, DHMIC was measured in four-day breast milk samples collected during the dietary record. Although only a single spot urine sample was used to determine UIC, urinary creatinine values were further assessed to adjust for urine dilution. To evaluate the iodine status of our donor population, we considered both UIC and DHMIC, as it has been argued that assessment based solely on UIC in lactating women can be misleading due to the fractional iodine excretion in breast milk and urine. In our study, with a median UIC of 112.41 µg/L and a median DHMIC of 148.48 µg/L we might assume an adequate iodine status of the recruited donors. Although there is currently no well-established cut-off point for setting iodine status by BMIC, in most studies the categorization of iodine status assessed by the median UIC was consistent with the categorization of iodine status assessed by the median BMIC cut off of 100 µg/L for determining iodine sufficiency in lactating women [30].

However, the study also presents limitations. The sample was not randomly selected; participation was voluntary and might have attracted donors more sensitive to their personal health and their children’s health and/or nutrition. Causal relationships could not be determined because of the cross-sectional design, even though the analysis was adjusted for different factors. Furthermore, we investigated iodine concentrations in non-pasteurized DHM. The effect of heat treatment on iodine concentration requires further investigation. However, pasteurization does not seem to influence iodine concentration [19]. On the other hand, milk was not pooled between donors or days, allowing us to establish relationships between DHMIC, iodine intake and a variety of other characteristics.

It is important to emphasize the unique composition and health benefits of MOM and DHM. Human milk not only provides macro- and micronutrients but also many other nutritional and functional properties important for growth and development, such as hormones, enzymes, antioxidants, growth factors, living cells, defensive elements, probiotics and likely many other components of which we know nothing about yet. Hence, if MOM or DHM fall short in meeting the special nutritional requirements of preterm infants, we need to be aware of it in order to improve and maintain the supply of human milk.

In conclusion, our study estimated a median DHMIC of 148.48 µg/L. Despite being an iodine-sufficient population, the milk of 70% of the donors studied did not reach the iodine concentration recommended to meet the needs of preterm infants. Iodine concentration in donated milk was higher in the supplemented donors. It would be interesting to study whether preterm infants who receive milk from iodine-supplemented donors obtain a clinical benefit.

## Figures and Tables

**Table 1:** Baseline characteristics of human milk donors (n = 113) and their breastfed infants.

	Median (p25, p75)
Age mother (years)	35.62 (32.89, 38.74)
Breastfeeding duration (month)	6.93 (4.70, 11.81)
Pre-pregnancy BMI (kg/m^2^)	22.07 (20.49, 24.80)
Current BMI (kg/m^2^)	22.79 (21.13, 25.03)
Gestational age (weeks)	39 (38, 40)
Birth weight (g)	3195.00 (2795.50, 3473.25)
	**n (%)**
Breastfeeding duration categories	
1–5 months	49 (43.36)
6–12 months	37 (32.74)
>12 months	27 (23.89)
Pre-pregnancy BMI categories	
<18.5	4 (3.54)
18.5–24.9	84 (74.34)
25–29.9	14 (12.39)
≥30	11 (9.73)
Current BMI categories	
<18.5	4 (3.54)
18.5–24.9	81 (71.68)
25–29.9	15 (13.27)
≥30	13 (11.50)
Breastfeeding ^a^	
Exclusive	51 (45.54)
Partial ^b^	61 (54.46)
Hypothyroidism during pregnancy	
Yes	16 (14.16)
Tandem breastfeeding	
Yes	6 (5.31)
Education level	
Secondary school and technical studies	16 (14.16)
University	97 (85.84)
Sex	
Girl	59 (52.21)
Boy	54 (47.79)
Children	
>1	50 (44.25)

^a^ n = 112 (due to one stillbirth), ^b^ Complementary food introduction.

**Table 2:** Donor human milk iodine concentrations (DHMIC) of study participants (n = 113) along with iodine intake variables, urine iodine concentration (UIC) and urine iodine creatinine ratio (UICR).

	Median (p25, p75)	Mean (±SD)
DHMIC (µg/L)	148.45 (97.61, 206.08)	163.36 (86.81)
Iodine intake (µg/day)		
From food alone	103.60 (86.45, 125.80)	111.35 (46.99)
Total intake ^a^	258.30 (153.70, 347.75)	254.6 (117.26)
UIC (µg/L)	112.41 (75.80, 160.10)	129.39 (73.76)
UICR (µg iodine/mg creatinine) ^b^	0.10 (0.07, 0.15)	0.11 (0.06)

^a^ Sample size regarding total iodine intake (considering information from diet, iodized salt and iodine supplement intake) was n = 84 (missing data for 29 donors as iodine supplement intake and/or type or salt consumed during the dietary record was not reported), ^b^ n = 112 (due to 1 missing data).

**Table 3:** Inadequacy of donor human milk iodine concentration (DHMIC) and iodine intake (n = 113).

	N (%)
Inadequate DHMIC for preterm infants (<200 µg/L)	79 (69.91)
Inadequate iodine intake from food alone according to	
Dold et al. (<167 µg/d) [27]	105 (92.92)
WHO (<250 µg/d) [28]	112 (99.12)
Inadequate total iodine intake ^a^ according to	
Dold et al. (<167 µg/d) [27]	24 (28.57)
WHO (<250 µg/d) [28]	39 (46.43)

^a^ Sample size regarding total iodine intake (considering information from diet, iodized salt and iodine supplement intake) was n = 84 (missing data for 29 donors as iodine supplement intake and/or type or salt consumed during the dietary record was not reported).

**Table 4:** Iodine-related characteristics of human milk donors (n = 113).

	n (%)
Iodine supplement use during pregnancy	
Yes	96 (84.96)
Partial	12 (10.62)
No	5 (4.42)
Iodine supplement use during dietary record *	
Yes	63 (55.75)
No	40 (35.40)
Missing data	10 (8.85)
Use of iodized salt during dietary record	
Yes	71 (62.83)
No	20 (17.70)
Missing data	22 (19.47)
Consumption of dairy products per day	
≥3 portion	40 (35.40)
<3 portion	73 (64.60)
Consumption of fish per week	
≥2 portion	61 (54.00)
<2 portion	52 (46.00)

* Daily dose of iodine supplement: median (p25, p75): 160 (120, 200) mcg/day, range: 40–200 mcg/day.

**Table 5:** Associations between donor human milk iodine concentration (DHMIC) and baseline characteristics, iodine intake variables, urine iodine concentration (UIC), urine iodine creatinine ratio (UICR) and iodine-related variables (univariate analysis).

	DHMIC (n = 113)	
	**rho**	***p*-Values**
**Characteristics (continuous variables)**		
Age mother (years)	−0.137	0.147
Breastfeeding duration (month)	−0.197	0.036
Pre-pregnancy BMI (kg/m^2^)	−0.000	0.997
Current BMI (kg/m^2^)	0.080	0.398
Gestational age (weeks)	−0.169	0.074
Birth weight (g)	−0.045	0.631
**Iodine Intake (µg/day)**		
From food only	0.046	0.628
Total intake ^a^	0.499	<0.000
**UIC (µg/L)**	0.059	0.535
**UICR (µg iodine/mg creatinine) ^b^**	0.175	0.064
	**Median (p25, p75)**	***p*-Values**
**Characteristics (categorical variables)**		
Breastfeeding duration		
1–5 months	158.30 (120.04, 205.43)	0.148
6–12 months	160.78 (92.61, 229.44)	
>12 months	111.03 (83.28, 194.35)	
Pre-pregnancy BMI categories		
<25	148.78 (95.45, 206.18)	0.744
25–29.9	164.61 (104.44, 229.97)	
≥30	141.85 (89.55, 232.03)	
Current BMI categories		
<25	148.45 (94.05, 205.43)	0.757
25–29.9	153.90 (128.13, 200.55)	
≥30	141.95 (95.53, 236.38)	
Breastfeeding ^c^		
Exclusive	153.30 (115.40, 205.88)	0.853
Partial ^d^	132.83 (87.84, 218,96)	
Hypothyroidism in pregnancy (yes)		
Yes	156.31 (99.89, 211.02)	1.000
No	145.77 (96.28, 206.27)	
Tandem breastfeeding		
Yes	203.21 (181.47, 331.68)	0.036
No	145.75 (95.18, 204.98)	
Education level		
Secondary school and technical studies	154.49 (128.35, 180.86)	0.651
University	145.75 (92.61, 206.30)	
Sex		
Girl	141.83 (87.63, 202.53)	0.289
Boy	158.40 (102.55, 208.96)	
Children		
1	145.75 (86.25, 200.50)	0.141
>1	157.09 (110.86, 209.55)	
**Iodine-related variables**		
Iodine supplement use during pregnancy		
Yes	147.10 (99.28, 205.65)	0.888
Partial	162.07 (100.31, 206.82)	
No	141.83 (63.82, 273.55)	
Iodine supplement use during dietary record		
Yes	182.70 (141.95, 229.15)	< 0.000
No	100.93 (70.88, 136.01)	
Use of iodized salt during dietary record		
Yes	158.30 (101.50, 205.88)	0.242
No	119.48 (82.78, 209.22)	
Consumption of dairy products per day		
≥3 portion	170.39 (129.58, 229.11)	0.044
<3 portion	135.32 (94.05, 199.90)	
Consumption of fish per week		
≥2 portion	153.90 (103.83, 206.10)	0.450
<2 portion	143.80 (86.70, 204.84)	

^a^ Sample size regarding total iodine intake (considering information from diet, iodized salt and iodine supplement intake) was n = 84 (missing data for 29 donors as iodine supplement intake and/or type or salt consumed during the dietary record was not reported), ^b^ n = 112 (due to 1 missing data), ^c^ n = 112 (due to one stillbirth), ^d^ Complementary food introduction.

**Table 6:** Coefficient B (95% CI) for donor human milk iodine concentration (DHMIC) by covariates (multivariate analysis).

Independent Variable ^a^	Model 1 (n = 112) ^b^	Model 2 (n = 103) ^c^
	Coefficient B (95%CI)	Coefficient B (95%CI)
Donor ID	−0.001 (−0.002–0.000)	0.000 (−0.002–0.001)
Breastfeeding duration (month)	**−0.010 (−0.016–−0.003)**	−0.004 (−0.011–0.003)
Gestational age (weeks)	−0.002 (−0.014–0.010)	−0.004 (−0.014–0.007)
Tandem breastfeeding (yes) ^d^	0.183 (−0.008–0.374)	0.132 (−0.048–0.313)
UICR (µg iodine/mg creatinine)	0.072 (−0.683–0.828)	−0.135 (−0.849–0.580)
Consumption of dairy products (≥3 portion per day) ^d^	0.071 (−0.024–0.166)	0.045 (−0.045–0.135)
Iodine supplement use during dietary record (yes) ^d^	-	**0.222 (0.135–0.310)**

CI: confidence interval, ID: identification number, UICR: urine iodine creatinine ratio, ^a^ DHMIC log 10 transformed, ^b^ Model 1: adjusted for Donor ID, breastfeeding duration, gestational age, tandem breastfeeding, UICR, consumption of dairy products per day, ^c^ Model 2: Model 1 and iodine supplement use during registration, ^d^ Reference categories (Coefficient B = 0): Tandem breastfeeding—no, Consumption of dairy products per day—<3 portion per day, Iodine supplement use during dietary record—no, remaining covariates are continuous variables, Values in bold: *p* < 0.05.

**Table 7:** Baseline characteristics, iodine intake, urine iodine concentration (UIC), urine iodine creatinine ratio (UICR) and iodine-related variables according to iodine supplement use during dietary record.

	Iodine Supplement Use during Dietary Record		*p*
	Yes (n = 63)	No (n = 40)	
	Median (p25, p75)	Median (p25, p75)	
**Characteristics (continuous variables)**			
Age mother (years)	34.91 (33.01, 38.45)	36.31 (32.56, 39.39)	0.570
Breastfeeding duration (month)	6.44 (4.40, 9.33)	9.46 (5.08, 17.01)	0.007
Pre-pregnancy BMI (km/m^2^)	21.96 (20.30, 24.91)	22.25 (20.36, 24.57)	0.789
Current BMI (km/m^2^)	23.07 (21.13, 25.29)	22.66 (20.66, 24.73)	0.725
Gestational age (weeks)	39 (37, 40)	39 (38, 40)	0.580
Birth weight (g)	3160.00 (2785.00, 3400.00)	3370.00 (2812.50, 3525.00)	0.203
**Iodine Intake (µg/day)**			
From food alone	103.60 (83.60, 132.25)	104.64 (83.02, 115.42)	0.365
Total intake ^a^	329.20 (278.15, 378.90)	143.30 (187.51, 91.74)	<0.000
**UIC (µg/L)**	119.05 (70.24, 160.50)	110.74 (87.97, 159.19)	0.995
**UICR (µg iodine/mg creatinine)** ^b^	0.116 (0.072, 0.153)	0.084 (0.061, 0.145)	0.109
	**n (%)**	**n (%)**	***p***
**Characteristics (categorical variables)**			
Breastfeeding duration			
1–5 months	29 (46.03)	15 (37.50)	0.011
6–12 months	25 (39.68)	9 (22.50)	
>12 months	9 (14.29)	16 (40.00)	
Pre-pregnancy BMI categories			
<25	49 (77.78)	32 (80.00)	1.000
25–29.9	7 (11.11)	4 (10.00)	
≥30	7 (11.11)	4 (10.00)	
Current BMI categories			
<25	46 (73.02)	32 (80.00)	0.620
25–29.9	9 (14.29)	3 (7.50)	
≥30	8 (12.70)	5 (12.50)	
Breast feeding ^c^			
Exclusive	32 (51.61)	14 (35.00)	0.109
Partial ^d^	30 (48.39)	26 (65.00)	
Hypothyroidism in pregnancy			
Yes	52 (82.54)	35 (87.50)	0.585
No	11 (17.46)	5(12.50)	
Tandem breastfeeding			
Yes	2 (3.17)	0 (0.00)	0.520
No	61 (96.83)	40 (100.00)	
Education level			
Secondary school or technical studies	10 (15.87)	4 (10.00)	0.558
University	53 (84.13)	36 (90.00)	
Sex			
Girl	27 (42.86)	26 (65.00)	0.043
Boy	36 (57.14)	14 (35.00)	
Children			
1	36 (57.14)	22(55.00)	0.841
>1	27 (42.86)	18 (42.86)	
BMIC ≥ 200 mcg/L			
Yes	29 (46.03)	3 (7.50)	<0.000
No	34 (53.97)	37 (92.50)	
**Iodine-related variables**			
Iodine supplement use during pregnancy			
Yes	56 (88.89)	31 (77.50)	0.313
Partial	5 (7.94)	6 (15.00)	
No	2 (3.17)	3 (7.50)	
Use of iodized salt during dietary record			
Yes	43 (68.25)	23 (57.50)	0.120
No	7 (11.11)	11 (27.50)	
Missing data	13 (20.63)	6 (15.00)	
Consumption of dairy products per day			
≥3 portion	26 (41.27)	10 (25.00)	0.137
<3 portion	37 (58.73)	30 (75.00)	
Consumption of fish per week			
≥2 portion	35 (55.56)	21 (52.50)	0.840
<2 portion	28 (44.44)	19 (47.50)	

^a^ Sample size regarding total iodine intake (considering information from diet, iodized salt and iodine supplement intake) was n = 84 (missing data for 19 additionally donors as the type of salt consumed during the dietary record was not reported), ^b^ n = 112 (due to 1 missing data), ^c^ n = 112 (due to one stillbirth), ^d^ Complementary food introduction. BMIC: breast milk iodine concentration.

## Data Availability

The data presented in this study are available on request from the corresponding author. The data are not publicly available due to privacy issues.
